# Assessing the effect of non-pharmaceutical interventions on COVID-19 transmission in Spain, 30 August 2020 to 31 January 2021

**DOI:** 10.2807/1560-7917.ES.2022.27.19.2100869

**Published:** 2022-05-12

**Authors:** David García-García, Rafael Herranz-Hernández, Ayelén Rojas-Benedicto, Inmaculada León-Gómez, Amparo Larrauri, Marina Peñuelas, María Guerrero-Vadillo, Rebeca Ramis, Diana Gómez-Barroso

**Affiliations:** 1 Consorcio de Investigación Biomédica en Red de Epidemiología y Salud Pública, CIBERESP, Madrid, Spain; 2 Centro Nacional de Epidemiología, Instituto de Salud Carlos IIII, Madrid, Spain; 3 Hospital Clínico San Carlos, Madrid, Spain

**Keywords:** COVID-19, Non-Pharmaceutical Interventions (NPIs), Spain, severity index

## Abstract

**Background:**

After a national lockdown during the first wave of the COVID-19 pandemic in Spain, regional governments implemented different non-pharmaceutical interventions (NPIs) during the second wave.

**Aim:**

To analyse which implemented NPIs significantly impacted effective reproduction number (R_t_) in seven Spanish provinces during 30 August 2020–31 January 2021.

**Methods:**

We coded each NPI and levels of stringency with a ‘severity index’ (SI) and computed a global SI (mean of SIs per six included interventions). We performed a Bayesian change point analysis on the R_t_ curve of each province to identify possible associations with global SI variations. We fitted and compared several generalised additive models using multimodel inference, to quantify the statistical effect on R_t_ of the global SI (stringency) and the individual SIs (separate effect of NPIs).

**Results:**

The global SI had a significant lowering effect on the R_t_ (mean: 0.16 ± 0.05 units for full stringency). Mandatory closing times for non-essential businesses, limited gatherings, and restricted outdoors seating capacities (negative) as well as curfews (positive) were the only NPIs with a significant effect. Regional mobility restrictions and limited indoors seating capacity showed no effect. Our results were consistent with a 1- to 3-week-delayed R_t_ as a response variable.

**Conclusion:**

While response measures implemented during the second COVID-19 wave contributed substantially to a decreased reproduction number, the effectiveness of measures varied considerably. Our findings should be considered for future interventions, as social and economic consequences could be minimised by considering only measures proven effective.

## Introduction

The rapid spread of the severe acute respiratory syndrome coronavirus 2 (SARS-CoV-2) virus and ensuing global coronavirus disease (COVID-19) pandemic forced governments worldwide to implement a wide variety of non-pharmaceutical interventions (NPIs), often with incomplete evidence of their effectiveness and with high social and economic expenses. The evolution of the disease and its interruption of daily life call for a rigorous analysis of the impact of public health measures in order to determine the most effective intervention.

With COVID-19 cases rising in Spain in early March 2020, the government established a state of emergency with the Royal Decree 463/2020 [1] on 14 March 2020, which triggered the introduction of NPIs aimed to slow the transmission of SARS-CoV-2. These included stay-at-home orders, limiting the movement of people, closure of restaurants and non-essential businesses and disinfection protocols among others; these measures were uniform throughout the country. Two months later, with the national order SND/399/2020 [2] on 9 May 2020, a de-escalation plan initiated the easing of certain restrictions. This procedure depended on the epidemiological status of each autonomous region, i.e. the political subdivisions of the territory, that were subject to less stringent regulations as their registered COVID-19 incidence decreased. The state of emergency ended on 21 June 2020, with mobility being restored throughout Spain and the autonomous regions regaining full authority over public health measures.

With the arrival of the second wave, the Spanish government enacted a second state of emergency on 25 October 2020 with the Royal Decree 926/2020 [3], in further attempts to contain the spread of the virus. Unlike the first state of emergency, it did not entail home confinement or specific restrictions for the whole country. Rather, the autonomous regions could adopt NPIs with different levels of stringency according to their own criteria and situation, within some established general categories. A clear example of these differences was the autonomous region of Madrid, which – in addition to measures applicable to its entire territory – designed a system of limitations by basic health zones, depending on the registered incidence of COVID-19 cases within the smaller administrative units [4].

Several studies have evaluated the impact of government policies on the evolution of the epidemic in Spain, with most focusing on the first wave [5-8]. While being a necessary exercise, the uniformity of the adopted measures and the presumably less reliable data sources available for this period because of the under-reporting during the initial stages of the pandemic may make these analyses inconclusive. A continued investigation of the subsequent waves is key for an informed response to possible future outbreaks of infectious diseases. 

The aim of this study was to measure the impact of NPIs on COVID-19 transmission in several different geographical regions in Spain from 30 August 2020 to 31 January 2021. We chose this time period in order to exclude the effect of COVID-19 vaccination from the analysis, as less than 1% of the population was vaccinated with a full primary course by 31 January [9]. We considered data from seven provinces and focused on the effective reproduction number (R_t_) as the main epidemiological indicator, which was particularly high during the first wave of the pandemic in Spain (estimated R_t_ was 3.56 (95% confidence interval (CI): 1.62–7.82) [10]).

## Methods

### Study setting

Spain is composed of 17 autonomous regions, subdivided into 52 provinces, and two autonomous cities. We focused our analysis on seven of these provinces: A Coruña, Barcelona, Madrid, Sevilla, València, Valladolid and Zaragoza, which together comprise 41% of the Spanish population [11]. These were chosen as a geographically and socially diverse sample of the country’s total population. The evolution of the number of COVID-19 cases across these regions during the time under study is available at https://cnecovid.isciii.es/covid19/#provincias.

### COVID-19 cases and effective reproduction number

COVID-19 cases, recorded by autonomous regions as part of the National Epidemiological Surveillance Network (RENAVE), are stored in the Spanish Surveillance System electronic platform (SiViES), and managed by the National Centre for Epidemiology. A COVID-19 case is considered confirmed by either a positive SARS-CoV-2 PCR test or an ELISA-based serological test (IgM) in patients with compatible symptoms and a negative PCR test; all confirmed cases are notified to RENAVE.

The R_t_ is the average number of secondary cases of disease caused by a single infected individual during the infectious period. This figure, which is time- and situation-specific, is commonly used to characterise pathogen transmissibility during an epidemic. We computed daily estimates for this parameter using the method of Cori et al. [12], implemented in the R package ’EpiEstim’, using a 7-day moving average (7-day window) in order to smooth possible notification delays.

### Non-pharmaceutical interventions

We reviewed the historical repositories [13-19] of the autonomous regions’ gazettes and recorded the activated NPIs, their levels of application, and the dates where any of these changed across the provinces under study. Several measures were excluded from the analysis, either because of high correlations with other variables (> 0.75: limited attendance to businesses and public infrastructures other than restaurant premises) or because they had either been widely implemented across Spain (mandatory use of masks) or not been sufficiently active across a large number of dates (< 10% of dates or only implemented in one province: local mobility restrictions, limited access to parks and green areas, restricted visits to nursing homes). The final dataset contained information concerning the levels of application of six NPIs: limited gatherings, curfews, regional mobility restrictions, mandatory closing times for public establishments, and limited seating capacity at bars and restaurant premises, both indoors and outdoors.

### Severity index

While there is no objective procedure to encode the level of stringency of any given public health measure, we proposed a universal scale for use in mathematical models. We computed a severity index (SI) for each of the NPIs included in the analysis as follows. We scaled linearly from 0 to 1 all the levels of application of any given measure, so that 0 models the absence of the restriction, and 1 models its most strict level of application, with equally spaced intervals between each level. For instance, in the period under study, mandatory curfews ranged hourly from 22:00 to 01:00. We thus assigned the values 0, 0.25, 0.5, 0.75, and 1 to the corresponding variable whenever there was no active curfew or curfews at 01:00, 00:00, 23:00, or 22:00, respectively. This is a rescaling of an integer-valued scale such as that used in some reference datasets [20-22], which yields common ranges of values (0–1) for all the measures. We also computed a global SI as the mean of the SIs of all the restrictions at each point in time; this is the natural choice of summary statistic as we assume linear effects for each of the measures in our analysis. Table 1 shows a detailed description of the levels of application of the six NPIs included in the analysis and their corresponding SI.

**Table 1:** Description of non-pharmaceutical interventions implemented during the second wave of the COVID-19 pandemic and the modelled severity index for their levels of application, Spain, 1 August 2020–31 January 2021 (n = 7 regions)

NPI	Levels of application			SI
Limited gatherings	No limitation			0
	15 people at indoor premises			0.125
	10 people at public spaces			0.25
	10 people, anywhere			0.375
	6 people, anywhere			0.5
	5 people, anywhere			0.625
	4 people, anywhere			0.75
	2 people, anywhere			0.875
	Only co-habitants			1
Curfew	No limitation			0
	01:00			0.25
	00:00			0.5
	23:00			0.75
	22:00			1
Regional mobility restrictions	No confinement			0
	Perimeter confinement of province/autonomous region			1
Mandatory closing times for non-essential businesses	No restriction			0
	After 23:00			0.2
	21:00–23:00			0.4
	19:00h–21:00			0.6
	17:00h–19:00			0.8
	Before 17:00			1
Limited indoor/outdoor seating capacity in bars and restaurant premises	No restriction	0	No restriction	0
	60 seats (indoors), 30 seats (outdoors)	0.25	75% of total capacity	0.125
			65% of total capacity	0.25
	25 seats	0.5	60% of total capacity	0.375
			50% of total capacity	0.5
	20 seats	0.75	40% of total capacity	0.625
			30% of total capacity	0.75
	Closed businesses	1	25% of total capacity	0.875
	2.5 m^2^ per seat	0.5	Closed businesses	1

COVID-19: coronavirus disease; NPI: non-pharmaceutical interventions; SI: severity index.

### Trend analysis

We performed a Bayesian change point analysis [23] on the R_t_ curves of each of the provinces under consideration. This method, implemented in the R package ‘bcp’ [24], computes a posterior probability for each point in a time series to have a change in mean, relative to other neighbouring points. This allowed us to distinguish points with high probability of a change in tendency – those which are expected to correspond to more abrupt intrinsic dynamics of the disease, e.g. peaks of the curve – from points with a slightly lower, nevertheless significant, probability – those that may be influenced by other factors, e.g. an increase in the SI.

### Statistical analysis

We quantified the possible effect of the NPIs under analysis using generalised additive models (GAMs) [25]. We first analysed the effect of the global SI on the R_t_ with a model given by the following formula:


$$R_{t}\;\sim \;s\left(time\right)+re\left(province\right)+global\;SI,$$

where *s(time)* models the smooth temporal component of the time series, the term *re(province)* incorporates the provinces as a random effect, and the global SI is assumed to be a linear covariate. We fitted several models with this structure to the data, allowing for a different number of basis functions in the construction of the temporal smooth (*k* thin plate splines, with *k* = 8,12,16,20,24), and chose the best model in Akaike’s Information Criterion (AIC) score [26] among them. In search of consistency, the process was repeated for 1-week-, 2-week- and 3-week-delayed R_t_ as the response variable, and we checked for improvement in estimation against a null model that had time as the only explanatory variable. We refer to these models as the ’global SI models’ below.

In case the global SI models yielded both a significant effect for the SI and an improvement in estimation compared with the null model, i.e. above 5% in deviance explained, we refined our approach by substituting the global SI in the previously fitted models by the covariates modelling a linear effect of the SIs of each of the restrictions described in Table 1. As before, we choose the best models in AIC for a 1 to 3 week-delayed R_t_, and we only accepted the models when there was a significant improvement in estimation compared with the global SI model, i.e. above 3% in deviance explained. We then identified which NPIs had a statistically significant effect in the model. We refer to these models as the ’individual SIs models’.

## Results

### Trend analysis

We attempted to identify possible associations between points with a mild probability for a change in mean with points where the global SI curve showed an increase or decrease (Figure). While some relationships between these two curves seemed to be present for some regions (Madrid and Valladolid), we were not been able to identify substantial associations consistently for any of the provinces.

**Figure:**
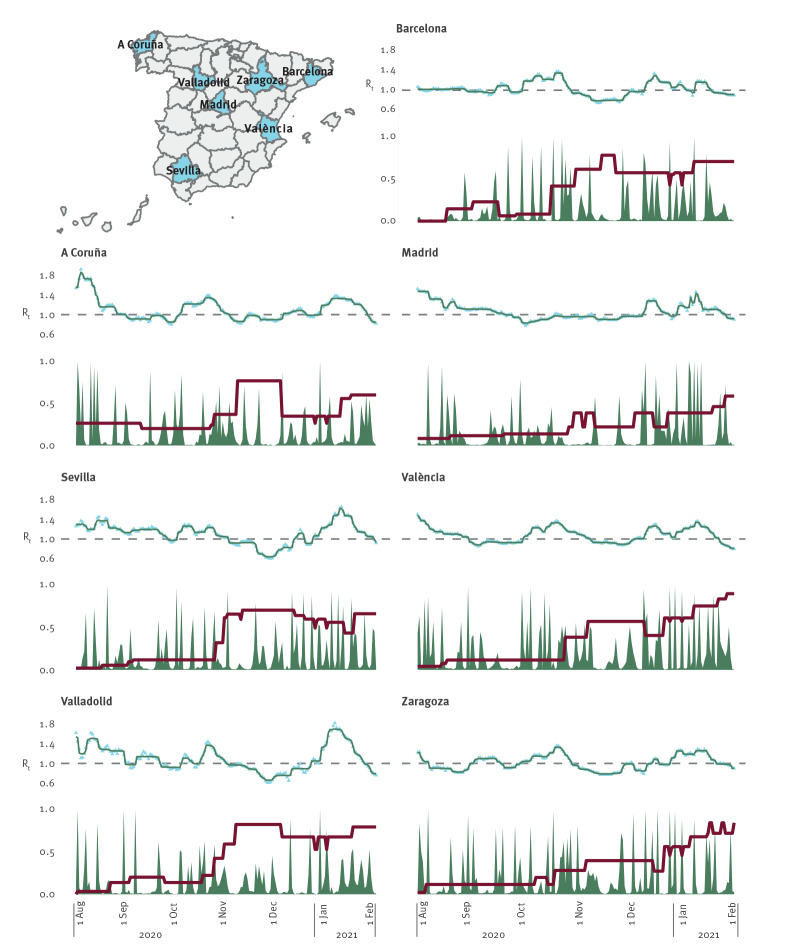
Observed effective reproduction number, results of the trend analysis and global severity index during the second wave of the COVID-19 pandemic, Spain, 1 August 2020–31 January 2021 (n = 7 regions)

COVID-19: coronavirus disease; R_t_: effective reproduction number.

For each of the seven provinces (blue areas on map insert), the top graph shows the observed effective reproduction number (R_t_, in blue) and its computed posterior mean (in green). Horizontal grey dashed line shows the R_t_ at 1.0 for reference. The bottom graph shows the posterior probability for a change in tendency for each point in time (in green) and the global severity index (in red).

### Statistical analysis

The global SI models provided a consistent improvement of the estimates when compared with the null model, and the global SI was a statistically significant covariate for 1-week-, 2-week- and 3-week-delayed R_t_. The R_t_ showed a decrease of 0.17 (95% CI: 0.11–0.23), 0.18 (95% CI: 0.13–0.23) and 0.12 (95% CI: 0.07–0.17) units per SI unit respectively for a 1-week, 2-week and 3-week delay in the observations. We obtained consistent results when assuming a non-linear effect of the global SI.

Table 2 shows the results of the statistical analysis of the individual SIs models. We found consistent results as well, with limited gatherings, mandatory closing times for non-essential businesses and limited outdoors seating capacity having a statistically significant lowering effect on the R_t_ for all the models (with average coefficients of −0.16, −0.14 and −0.11, respectively). Curfews had an increasing effect on the 2-week- and 3-week-delayed R_t_, while regional mobility restrictions and limited indoors seating capacity were not significant covariates in any of the fitted models.

**Table 2:** Linear effects for non-pharmaceutical interventions yielded by the individual severity index models during the second wave of the COVID-19 pandemic, Spain, 1 August 2020–31 January 2021 (n = 7 regions)

Response variable	Variable	Linear effect	95% CI
1-week-delayed R_t_	Mandatory closing times	−0.16	± 0.06*
	Limited gatherings	−0.14	± 0.07*
	Limited outdoor seating capacity	−0.1	± 0.04*
	Curfew	0.01	± 0.04
	Regional mobility restrictions	−0.01	± 0.02
	Limited indoor seating capacity	0.01	± 0.03
2-week-delayed R_t_	Mandatory closing times	−0.15	± 0.06*
	Limited gatherings	−0.18	± 0.06*
	Limited outdoor seating capacity	−0.12	± 0.04*
	Curfew	0.05	± 0.03*
	Regional mobility restrictions	−0.02	± 0.03
	Limited indoor seating capacity	0	± 0.04
3-week-delayed R_t_	Mandatory closing times	−0.1	± 0.05*
	Limited gatherings	−0.16	± 0.06*
	Limited outdoor seating capacity	−0.12	± 0.04*
	Curfew	0.04	± 0.03*
	Regional mobility restrictions	0	± 0.02
	Limited indoor seating capacity	0	± 0.04

CI: confidence interval; COVID-19: coronavirus disease; R_t_: effective reproduction number.

Statistically significant variables (p value < 0.05) are indicated with an asterisk.

We obtained consistent results when removing highly correlated variables from the dataset. More precisely, we selected the two pairs of variables that showed the highest correlation – regional mobility restrictions and curfews: 0.8 pair-to-pair correlation and limited indoors and outdoors seating capacity: 0.78 pair-to-pair correlation, removed any of these four variables from the dataset and confirmed that the results of the statistical analysis were consistent. The remaining pair-to-pair correlations were < 0.55.

## Discussion

We have evaluated the impact on the R_t_ associated with various NPIs implemented in Spain by the governments in the autonomous regions during the second wave of the COVID-19 pandemic. While we identified a general lowering effect of the NPIs on R_t_, our analysis suggests that some measures were more effective than others, in agreement with other studies on the topic [27,28]. Namely, the limitation of public and private gatherings, mandatory closing times for non-essential businesses and restricted maximum seating capacity in outdoor premises had a consistent decreasing effect on the R_t_. We found that regional mobility restrictions and limitations on indoors seating capacity did not have a statistically significant effect on the R_t_, while curfews contributed to a slight increase in R_t_.

While current knowledge has identified indoor facilities as a suitable source of infection [29], the statistically significant result found instead for restricted maximum seating capacity in outdoor premises may be due to the high correlation between these two covariates (0.78). An indication favouring this interpretation is the fact that restricted indoor seating capacity becomes a significant variable with a lowering effect on the R_t_ when removing indoor restrictions from the analysis. Another unexpected result is that curfews contributed positively to the virus transmission; this could be a consequence of the behaviour patterns of the population who may resort to home gatherings during times when curfews are active thus increasing the probability of infection.

Several reference data sources record NPIs and their levels of stringency by means of a continuous index, rather than a discrete variable [20-22], an approach which we adopted in the present study. Nevertheless, most of the studies concerned with a qualitative assessment of the effect of public health measures on the COVID-19 curve have exploited a simpler formulation in terms of binary variables, indicating only whether a given measure was active or not at any point in time [27,28,30]. We replicated our statistical analysis following this method, replacing the global SI with the number of active measures, but we obtained non-significant results that were not consistent under perturbations of the models (data not shown). We, thus, presume that continuous indexes such as the ones used here may be a useful tool for future investigations. From the epidemiological point of view, we believe that continuous indexes can also better reflect the interactions between different constraints, as these are usually implemented at the same time and thus some strategy is needed to isolate their individual effects.

We did not obtain any clear interpretations from the trend analysis. After examination of the resulting curves, we are led to think that the disease has a strong intrinsic tendency, and that the particular socioeconomic, cultural, and geographic differences (among others) across provinces make the comparisons between them non-significant. This is consistent with other studies that have identified the high variability in the effectiveness of public health measures [31-38]. The results of the statistical analysis seem to contribute to this hypothesis, as a similar analysis for other epidemiological indicators that are usually more subject to inertia (14-day cumulative incidence, number of hospitalisations, number of deceased) yielded inconsistent results (data not shown).

### Limitations

Among the several factors that could limit our approach, one intrinsic to our setting is the high correlation between the variables that model the NPIs (0.47 average pair-to-pair correlations). Indeed, most of these measures were implemented simultaneously, often with a parallel change in level of stringency, a fact that may distort the statistical outcome and complicate the evaluation of single measures. Other relevant limitations common to studies on effectiveness of NPIs [27,28,36-38] include the lack of a ‘control group’ of regions without any active NPIs, and the fact that NPIs are usually enforced as a response to the increased severity of the epidemic, which may result in a confounding effect for the statistical models. We hoped to mitigate this effect by the validations and controls for consistency incorporated in our model fitting and selection process. For the same reason, since the use of face masks was mandatory nationwide since the start of the pandemic in Spain, our approach is unable to capture the effect of masking on the transmission of the disease. Finally, the difficulty to measure the actual adherence to public health policies may also restrict the scope of our analysis. While some studies address the understanding of the factors that determine it [39] and the general perception of the pandemic in Spain [40], no real data are available to verify if the computed SIs actually reflect the population’s behaviour during the time of study.

## Conclusions

Our analysis suggests that the NPIs implemented during the second wave of COVID-19 in Spain had a significant impact in the spread of the disease, with some measures being more effective than others, including limited gatherings, mandatory closing times for non-essential businesses, and restricted outdoors seating capacity. Nevertheless, the epidemic curve appears to have a strong intrinsic trend that requires an informed and context-dependent perspective for an effective control of the pandemic.
